# Assessment of the sexually abused female children admitted to a tertiary care hospital: Eight year experience

**DOI:** 10.12669/pjms.305.5274

**Published:** 2014

**Authors:** Leyla Mollamahmutoglu, Ozlem Uzunlar, Inci Kahyaoglu, Sebnem Ozyer, Mustafa Besli, Mujdegul Karaca

**Affiliations:** 1 Leyla Mollamahmutoglu, Obstetrics and Gynecology Specialist, Department of Obstetrics and Gynecology, Etlik Zubeyde Hanim Women’s Health Research and Education Hospital, Ankara, Turkey.; 2 Ozlem Uzunlar, Obstetrics and Gynecology Specialist, Department of Obstetrics and Gynecology, Zekai Tahir Burak Women’s Health Research and Education Hospital, Ankara, Turkey.; 3 Inci Kahyaoglu, Obstetrics and Gynecology Specialist, Department of Obstetrics and Gynecology, Etlik Zubeyde Hanim Women’s Health Research and Education Hospital, Ankara, Turkey.; 4 Sebnem Ozyer, Obstetrics and Gynecology Specialist, Department of Obstetrics and Gynecology, Zekai Tahir Burak Women’s Health Research and Education Hospital, Ankara, Turkey.; 5 Mustafa Besli, Obstetrics and Gynecology Specialist, Department of Obstetrics and Gynecology, Zekai Tahir Burak Women’s Health Research and Education Hospital, Ankara, Turkey.; 6 Mujdegul Karaca, Obstetrics and Gynecology Specialist, Department of Obstetrics and Gynecology, Zekai Tahir Burak Women’s Health Research and Education Hospital, Ankara, Turkey.

**Keywords:** Childhood, Incest, Sexual abuse

## Abstract

***Objective:*** To discuss the medical, social and legal characteristics of the child sexual abuse and to provide a perspective for gynecologists on this topic.

***Methods: ***A retrospective analysis was carried out of the medicolegal records of female children below the age of 18 referred to a tertiary teaching hospital and diagnosed as being exposed to sexual abuse within the family between the years of 2004 to 2012.

***Results: ***One hundred and thirty-nine cases were diagnosed as being exposed to sexual abuse during the 8 year period, 23 of them (16.5%) had been involved in sexual abuse within the family. Eleven out of 23 had been admitted as part of a legal process while the rest were reported by a third person.

***Conclusion: ***Since sexual abuse within the family is a taboo in Islamic societies, the diagnosis **can** take a long time. Recognition of sexually abused children, providing early performance of medicolegal examinations, and applying standardized medical guidelines are essential to protect these children.

## INTRODUCTION

The childhood years of human life area sensitive period that is supposed to be carefree time of physical and psychosocial development. Child sexual abuse (CSA) is considered one of the greatest threats to a child’s well-being and safety. Children and adolescents who have been sexually abused can suffer from a range of mild to severe psychological and behavioral problems, in both the short and long term including depression, substance abuse, eating disorders, premenstrual syndrome, sexual disorders, multiple personality disorders, adjustment disorders, somatoform disorders, and posttraumatic stress disorders.^1^ CSA is a far-reaching and complex problem with physical, emotional, ethical, cultural, and legal aspects. CSA is not a novel happening, it has existed over centuries; however, its clinical importance and handling as a pediatric problem have expanded rapidly in recent years. What Kempe called a ‘hidden pediatric problem’ in 1977 is certainly less hidden at present.^2^ Despite the increased public awareness, which has led to greater reporting, CSA still remains under-reported because of the shame and guilt caused by the abuse. Unfortunately, it is estimated that less than 35% of all incidents of CSA are being identified by childcare professionals.^3^ Thus, it may be thought that number of cases represent the tip of the iceberg. WHO estimates that globally some 40 million children aged 10-14 years suffer from some form of abuse and neglect requiring health and social care.^4^ Figures from the United States show that 1 in 4 girls and 1 in 6 boys are sexually abused before the age of 18, whereas the median age for reported abuse is 9 years old.^5^^,^^6^

Different definitions of sexual abuse have been employed. The National Center on Child Abuse and Neglect defines sexual abuse as ‘contact or interaction between a child and an adult, when the child is being used for the sexual stimulation of that adult or another person’.^7^Sexual abuse may also be committed by another minor, when that person is either significantly older than the victim (defined as more than 5 years), or when the abuser is in a position of power or control over the child.^7^

The purpose of this study was to investigate the epidemiology of CSA, the characteristics of the perpetrators, to review social, medical and legal aspects of the situation that a gynecologist faces when managing a child with suspected sexual abuse, to discuss the treatment strategies and to help establish an appropriate medical and legal approach.

## METHODS

This retrospective study was performed in the Zekai Tahir Burak Women’s Health Research and Education Hospital, which is a tertiary care hospital with 545 beds and 20,000 deliveries per year, that is located in Ankara, the capital city of Turkey. As a hospital policy, cases of childhood sexual abuse and neglect are handled by a multidisciplinary team providing full assessment and treatment. This team consists of gynecologists, psychiatrists, dermatologists, psychologists, dieticians, social workers, and nurses.

***Clinical evaluation:***

- ***Interview:*** A comprehensive interview was conducted by one of the gynecologist or psychiatrists who are specialized in adolescents. When possible, the parent or the accompanying person was not present during the interview so that influences and distractions were kept to a minimum. Intra-familial relations, social environment, relations with friends, characteristics of school and education were evaluated. A history was taken from the accompanying person as well.
- ***Psychiatric evaluation:*** The effects of the trauma were investigated. The alterations in sleep and appetite, school status, alterations in social life and relations with peers were questioned.
- ***Physical examination and forensic investigation:*** Since the children could be anxious about being examined, the examination was explained before being performed. Top-to-toe physical examination was performed, which included careful assessment for signs of physical abuse such as injuries and bruises or self-injurious behaviors. Specific attention was given to the sexual development of the areas involved in sexual activity, including the anogenital region. The next concern was the collection of biologic forensic evidence. For sperm identification, swabs were collected from the hymenal ring, vagina, and the anus when the sexual abuse occurred within 72 hours. Cultures and serologic tests for STD were collected depending on the history of abuse. Pregnancy test and ultrasonography were performed to detect a possible pregnancy. All findings were recorded in the file of the patient.

A retrospective study was conducted based on the review of these medicolegal records of female children below the age of 18 who were evaluated for apparent or suspected sexual abuse between the years of 2004 and 2011. The study was approved by the local ethics committee of the hospital. Demographic data, child and family characteristics, physical and genital findings, and interventions were noted.

## RESULTS

One hundred and thirty nine cases were diagnosed as being exposed to sexual abuse during the 8 year period, 23 of them (16.5%) had been involved in sexual abuse within the family. One hundred fourteen out of 139 cases (82%) had been admitted as a part of a legal process. The rest of the abused children were reported by a third person usually the mother or grandmother or suspected by the gynecologists working in the adolescent department (Table-I). The ages of the children ranged from 2 to 23 years and the mean age of the victims was 15.3±2.3 years. Two of them were preschool children, 6 were in primary school, 84 in intermediate and high school, and 47 of them were not attending any educational institution. The parents of 43.9% of the victims were divorced. Low socioeconomic status was documented in 73.4% of the families. Acute genital injury occurred in 12.2% of the girls and 2.9% of them needed surgical intervention (Table-II). Vaginal penetration was the type of abuse in 71.2% of the victims and genital touching and fondling in 28.8% of the cases. Anal penetration was reported in 10.1% of the cases. In 58.3% of the cases in which acute sexual assault or sexual abuse occurred within 72 hours, swabs were taken for detection of spermatozoa. No spermatozoa could be detected in vaginal and anal swabs of these cases. About 16.5% of the victims had evidence of physical trauma. In 43.2% of the cases, the perpetrator was known to the victim, while in 56.8% of cases, a stranger was the perpetrator and in 23 cases, the perpetrator was a family member. In 46.8% of children, abuse occurred only once, however the abuse occurred on multiple occasions in 48.2% of the children. 58 pregnancies were detected. None of the cultures or serologic tests revealed positive results for sexually transmitted diseases (STD), which is routinely performed for all children.

## DISCUSSION

CSA is a devastating event for a child, which is kept as a secret until adulthood most of the time, because of the shame, guilt and fear of punishment or abandonment. Patients may not identify themselves as survivors of sexual abuse. Even if mothers are aware of incestuous events to which their children are exposed, only 3% of them attempt to remove the child from the incestuous environment, such as taking them away from home, divorcing to obtain their child’s parental rights, or complaints to authorities for criminal prosecution. The sexual abuse usually becomes evident when the child is physically hurt. Thus, it is hard to determine the actual number of cases. Studies have stated that CSA is far more frequent than all malignancies in childhood.^8^^,^^9^ In the study of Al-Mahroos et al, it was reported that there had been a 2.5% increase in the reported cases of CSA during the years of 2000-2009.^10^ In 2002, more than 88,000 children were confirmed victims of sexual abuse in the United States.^11^ Studies have suggested that each year approximately 1% of children experience some form of sexual abuse, resulting in the sexual victimization of 12% to 25% of girls and 8% to 10% of boys by 18 years of age.^11^ It is reported that girls are four times more likely to be abused than boys.^12^ Fifty-three percent of the victims are reported to be below 14 years of age.^13^ Despite an increase in the rate of CSA, the rate of referral to court and the rate of sentencing is low.^12^ In a study involving 118 CSA cases conducted in England, the rate of referral to court and the rate of sentencing was reported to be 36% and 17%, respectively.^14^ In another study from Mexico, the rate of referral to legal authorities was reported as 3.7%.^7^

**Table-I:** Socio-demographic characteristics of the victims of childhood sexual abuse

	***Frequency (n,%)***
Age at abuse (years)	15.3±2.3*
***Education***	
Preschool Primary school Intermediate to high school Not attending any educational institution	2 (1.4%) 6 (4.3%) 84 (60.5%) 47 (33.8%)
***Marital status of the parents***	
Divorced Living together	61(43.9%) 78 (56.1%)
***Socioeconomic level***	
Low income Medium-high income	102 (73.4%) 37 (26.6%)
***Is the abuser known to child?***	
Yes No	60 (43.2%) 79 (56.8%)

* Mean±Standart deviation.

**Table-II:** Clinical presentation of childhood sexual abuse victims to the hospital

	***Frequency (n,%)***
Acute genital injury	17 (12.2%)
Surgical intervention required for genital injury	4 (2.9%)
***Type of sexual abuse***	
Vaginal penetration Genital touching and fondling	99 (71.2%) 40 (28.8%)
Presence of anal penetration	14 (10.1%)
Evidence of physical trauma	23 (16.5%)
Unwanted pregnancy	58 (41.7%)

Children of all ages may be sexually abused. CSA peaks at a mean age of 8-12 years and 30-50% of abuse cases take place outside the family, by either an acquaintance or a total stranger.^15^ In two recent studies carried out in Turkey, 40.7-66.7% of abusers were found to be known to the children.^16^^,^^17^ In the majority of the cases (43.2%) included in our study, children were mostly abused by individuals who were known to them.

An important subgroup of sexual abuse is incest. It may be defined as sexual relations between close blood relatives, e.g., between a child and the father or uncle, between siblings; or in its broader sense, between a child and a stepparent or stepsibling.^18^ In the study of Raboei et al in which 78 CSA patients including boys and girls were enrolled, the incidence of intra-familial abuse was reported to be as high as 53.8%.^19^ Csorba et al. in their study of female child sexual abuse, found that in one-fifth of cases, the person accused of abuse was a family member.^20^ The results of the present study revealed that 16.5% of CSA was intra-familial sexual abuse. This is of particular interest because of two factors; first there is a greater degree of conspiracy surrounding the problem, thus making it harder to expose than other forms of sexual abuse. Second, there is evidence that the likelihood of serious long-term sequelae is greater for incest victims than for victims of other forms of abuse.^21^

Although CSA is present in all kind of socioeconomic groups, studies have shown that more severe forms of abuse appear to be associated with lower socioeconomic status.^18 ^The divorce rate and low socioeconomic status among the children’s parents in the study were found to be as 43.9% and 73.4% respectively. Low socioeconomic status was over-represented among the families in this study. These observations suggest that lower socioeconomic status and divorce might be risk factors for sexual abuse during childhood.

Certain signs and symptoms should immediately alert a physician to the probability that a child is an abused victim; trauma to the genital area, presence of sexually transmitted disease (STD), chronic vulvovaginitis, abnormal findings on genital examination, pregnancy, symptoms of emotional stress, and reports of sexualized behaviour inappropriate for the child’s age and development, e.g., drawings, statements, or sexualized play.^22^ In our study,17 (12.2%) cases were presented with an acute trauma to the genital area. 58 pregnancies were detected but no sexually transmitted disease was diagnosed. But in previous reports, STD was reported to be as high as approximately 5%.^23^

An extensive list of sequelae are attributed to sexual abuse. Depression, anxiety, phobias, substance abuse, psychosis, suicidal thoughts or statements, sexual dysfunction, somatization, multiple personality disorder, posttraumatic stress disorder, anorexia nervosa, problems with trust, borderline personality disorder, parenting difficulties and risk of own child being abused were also reported as sequelae of CSA.^22^

In conclusion, although both the children and their families keep it as a secret, CSA is a serious condition that needs urgent evaluation as it is suspected or diagnosed. One of the most important members of the evaluating team is the gynecologist, who usually has the initial encounter with these children.

## Authors Contribution:

**LM, OU, IK, SO:** Designed the study, did the literature search.

**LM, OU, IK, SO, MB, MK:** Conducted the study, did data analysis.

**LM, OU, IK, SO:** Prepared the final draft for publication.
